# Supplementary material for: Cloud BioLinux: pre-configured and on-demand bioinformatics computing for the genomics community
Source: BMC Bioinformatics. 2012 Mar 19;13:42. doi: 10.1186/1471-2105-13-42 (PMC3372431; doi:10.1186/1471-2105-13-42)
Supplement: Additional file 1 — Supplementary 1 Cloud BioLinux software documentation in the form of a mini, self-contained website. Users need to download and uncompress the .zip file, and open through a web browser the "index.html" file available on the main directory. (ZIP 1823 kb). [file 1471-2105-13-42-S1.ZIP › Cloud-BioLinux-Package-Documentation/docs/gff2ps.html]

Bio-Linux Software Documentation Pages

Back to search form

## gff2ps

|  |  |
| --- | --- |
| Name | gff2ps |
| Description | gff2ps is a script program developed to convert gff-formatted records into high quality one-dimensional postscript plots. Such plots maybe useful for comparing genomic structures and to visualise outputs from genome annotation programs.  The gff2ps package is prepared by Debian-Med and can be installed in Bio-Linux by typing: `sudo apt-get install gff2ps` For further information about gff2ps please see the remote documentation link |
| Homepage | http://genome.crg.es/software/gfftools/GFF2PS.html |
| Remote Documentation | http://genome.imim.es/software/gfftools/gff2ps\_docs/visualizing/index.html |
